# Insulin resistance exhibits varied metabolic abnormalities in nonalcoholic fatty liver disease, chronic hepatitis B and the combination of the two: a cross-sectional study

**DOI:** 10.1186/s13098-019-0440-z

**Published:** 2019-06-15

**Authors:** Junzhao Ye, Xuan Hu, Tingfeng Wu, Yanqin Wu, Congxiang Shao, Fuxi Li, Yansong Lin, Shiting Feng, Wei Wang, Bihui Zhong

**Affiliations:** 1 Department of Gastroenterology, The First Affiliated Hospital, Sun Yat-sen University, No. 58 Zhongshan II Road, Yuexiu District, Guangzhou, 510080 China; 2 Department of Radiology, The First Affiliated Hospital, Sun Yat-sen University, No. 58 Zhongshan II Road, Yuexiu District, Guangzhou, 510080 China; 3 Department of Ultrasound, The First Affiliated Hospital, Sun Yat-sen University, No. 58 Zhongshan II Road, Yuexiu District, Guangzhou, 510080 China

**Keywords:** Nonalcoholic fatty liver disease, NAFLD, Chronic hepatitis B, CHB, Insulin resistance, IR, Metabolic symptoms

## Abstract

**Background:**

Insulin resistance (IR) related metabolic disorders are associated with a worse prognosis of chronic hepatitis B virus (CHB) infection or nonalcoholic fatty liver disease (NAFLD). However, the relationships among CHB, steatosis, IR and metabolic factors remain controversial. The study aims to evaluate the impact of insulin resistance severity on metabolic profiles in patients with CHB, NAFLD and the coincidence of the two.

**Methods:**

We conducted a cross-sectional study between January 2011 and December 2018 that included 2768 consecutive Chinese subjects (healthy controls: 667, CHB: 970, NAFLD: 878, CHB with NAFLD: 253). IR was determined with the homeostasis model assessment for insulin resistance (HOMA-IR). Metabolic measures included fasting serum insulin, glucose, lipid profiles and uric acid.

**Results:**

The prevalence of IR was increased in CHB with NAFLD subjects compared with that in control subjects or subjects with CHB or NAFLD alone (41.5% vs 2.9%/11.9%/36.9%, respectively; *P *< 0.001). Within NAFLD and CHB with NAFLD group, the frequency of metabolic syndrome, hypertension and hyperuricemia increased as the HOMA-IR category increased (*P* for trend < 0.05). A higher risk for total cholesterol, low-density lipoprotein cholesterol and elevated alanine transaminase was observed with IR in the CHB with NAFLD group compared with that in the other groups, while no stepwise increase in hypertriglyceridemia was found in HOMA-IR in the CHB with NAFLD group.

**Conclusion:**

Insulin resistance is highly prevalent in patients with CHB combined with NAFLD, and the increased metabolic risk, rather than hypertriglyceridemia, is driven by IR in CHB combined with NAFLD.

## Background

Nonalcoholic fatty liver disease (NAFLD) and chronic hepatitis B (CHB) are the two most common types of liver diseases worldwide. The growing epidemic of NAFLD has reached over 26% of the world’s population [1], and chronic hepatitis B virus (HBV) infection affects over 300 million patients [2]. Notably, their overlap is also common, with a prevalence of up to 25–30% in CHB [3].

Metabolic risk factor burden, including abdominal obesity, hypertension, dyslipidemia, diabetes mellitus and hyperuricemia, has been verified to strongly affect the progression and even all-cause mortality of CHB and NAFLD [4, 5]. The constellation of metabolic disturbances may be more crucial than single components of metabolic syndrome in determining the long-term prognoses of both diseases. Therefore, the focus on the relationship among metabolic disturbance, HBV infection and NAFLD is growing. NAFLD predisposes individuals to the development of all metabolic comorbidities, whereas meta-analysis shows that patients with CHB have reduced risks of metabolic syndrome or some of its components compared to the general population [6]. However, in the context of complex interactions where hepatic steatosis and virologic backgrounds play opposite roles, the metabolic derangement effect of NAFLD on CHB remains unclear.

Insulin resistance (IR) is acknowledged as the physiological basis of metabolic syndrome and NAFLD and is characterized by an impairment of insulin-mediated glucose utilization in target tissues [7]. In insulin-resistant states, disrupted glucose homeostasis induces excess lipid substrate deposition in the liver, which is involved in the liver fibrosis progression and development of hepatocellular carcinoma of CHB [5, 7]. Moreover, increasing evidence indicates that IR and associated diabetes are overrepresented in CHB [8–11]. For example, an increased association was observed between maternal HBsAg carrier status and risk development of gestational diabetes in a retrospective cohort [9]. Lu et al. also showed an increased frequency of HBV infection in type 2 diabetic patients but not in patients with adult-onset autoimmune diabetes [10]. Another cross-sectional study with a large sample size demonstrated that CHB status was independently associated with IR defined by homeostasis model assessment (HOMA) criteria or a quantitative insulin check index [12]. As noted, superimposed IR does not increase in parallel with metabolic syndrome in CHB patients, unlike NAFLD with IR, which increases the risk of metabolic abnormalities. This evidence suggests that IR may mediate distinct metabolic profiles in CHB with or without NAFLD. However, whether different types of liver diseases have the same metabolic consequences as the degree of IR increases or whether all of these metabolic components should be monitored aggressively for those patients with IR remains unclear.

Given the high prevalence of IR, metabolic syndrome, NAFLD and CHB, clarifying their associations plays an important role in developing screening strategies, especially for identifying patients with higher risks of specific combinations of metabolic complications at various levels of IR severity. The aims of the study were to assess the influence of IR severity measured by the HOMA on the risks of metabolic factors in subjects with NAFLD, CHB and CHB with NAFLD.

## Materials and methods

### Study population and design

This was a cross-sectional study of a single-center cohort conducted at the Hepatology Outpatient Clinic and health examination center of the First Affiliated Hospital of Sun Yat-sen University, China. We consecutively enrolled subjects from January 1, 2011 to December 31, 2018 with the following inclusion criteria: (1) patients aged 18–65 years; (2) complete anthropometry, abdominal ultrasound, and laboratory results; (3) diagnosis of chronic hepatitis B and/or nonalcoholic fatty liver disease; (4) all patients were naïve to antiviral therapy or treatment for metabolic diseases. CHB was diagnosed in patients who were positive for hepatitis B surface antigen (HBsAg) for over 6 months, and the diagnosis of NAFLD was established by abdominal ultrasonography.

The exclusion criteria included: (1) healthy individuals with any medical history of severe illness or prescription indicating the presence of a chronic illness; (2) subjects taking nutritional supplements of any sort; (3) the imaging evidence of hepatocellular carcinoma (computed tomography [CT] or magnetic resonance imaging [MRI] scan of the abdomen) and the level of AFP, other end-stage liver diseases, hepatitis C virus infection (tests for antibody against hepatitis C virus), autoimmune liver disease (tests for anti-nuclear antibody, anti-smooth muscle antibody and anti-mitochondrial antibody); (4) diabetes mellitus or other endocrine diseases; (5) pregnancy and breastfeeding; (6) patients with significant fibrosis detected with liver stiffness measurement (LSM) by real-time shear wave elastography or (7) previous history of alcohol consumption of > 140 g/week in men or > 70 g/week in women. The Clinical Research Ethics Committee of The First Affiliated Hospital of Sun Yat-Sen University approved the study protocol, and all subjects provided written informed consent.

### Clinical evaluation

Patient history, including demographics, past disorders, medication history and nicotine and alcohol consumption, were collected with a questionnaire interview. All subjects underwent anthropometric measurements, including body weight, body height, waist circumference, hip circumference and blood pressure. Body mass index (BMI) was defined as the body weight in kilograms divided by the square of the body height in meters. Waist circumference was measured in centimeters at the midpoint between the lower margin of the rib cage and the top of iliac crest using anon elastic measuring tape, and hip circumference was also measured in centimeters at the widest point between the hip and buttock using the same tape. The waist-to-hip ratio (WHR) was calculated by dividing the waist circumference by the hip circumference.

### Assessment of insulin resistance status (exposure)

Fasting blood glucose (FBG) and serum insulin (FINS) (both measure after fasting for over 8 h) were measured by an Abbott c8000 Automatic Biochemistry Analyzer (Abbott, USA). The HOMA of the IR index was calculated using the following equation: HOMA-IR = FINS (µU/mL) * FBG (mmol/L)/22.5 [13].

### Measurements of metabolic profiling (outcomes)

Sitting blood pressure was measured twice by physicians using an Omron (J710, Japan) electronic monitor applied to the right upper arm after a 15-min rest. Biochemical parameters were assayed as mentioned previously, including total cholesterol (CHOL), triglycerides (TG), high-density lipoprotein cholesterol (HDL-C), low-density lipoprotein cholesterol (LDL-C), uric acid (UA), albumin (ALB), total bilirubin (TB) and liver enzymes [alanine aminotransferase (ALT), aspartate aminotransferase (AST)].

We defined obesity as BMI ≥ 25 kg/m^2^ [14], and metabolic syndrome was diagnosed according to the modified criteria for an Asian population [15]. Hypertension was diagnosed in subjects with systolic blood pressure (SBP) ≥ 140 mmHg or diastolic blood pressure (DBP) ≥ 90 mmHg or previously diagnosed hypertension [16]. Hypercholesterolemia was defined as a total CHOL level > 5.2 mmol/L. Hypertriglyceridemia was defined as a TG level > 1.7 mmol/L. A low HDL-C level was defined as an HDL-C level < 1.0 mmol/L. A high LDL-C level was defined as an LDL-C level > 3.4 mmol/L [17]. Hyperuricemia was diagnosed in males and females at > 420 and 360 µmol/L, respectively [18]. The normal upper limit for ALT and AST were set to 40 and 37 U/L, respectively.

### Radiology examination

Fatty liver was assessed by abdominal ultrasonography in all subjects using criteria as the presence of liver and kidney echo discrepancy, with or without the presence of posterior attenuation of ultrasound beam, vessel blurring, difficult visualization of the gallbladder wall, difficult visualization of the diaphragm. Liver fat content was further assessed using MRI fat signal fraction by two-point DIXON-fat–water-separation MRI at 3.0 Tesla (SIEMENS 3.0T MAGNETOM Verio). The scanning protocol and imaging parameters were described in detail in our previous study [19]: TE1 2.5 ms; TE2 3.7 ms; repetition time 5.47 ms; 5° flip angle; ± 504.0 kHz per pixel receiver bandwidth; and a slice thickness of 3.0 mm. Fat content was calculated using an irregularly shaped region of interest (ROI) covering the entire liver in 21 consecutive slices (maximum-area centered) for each patient. The liver fat content was classified by MRI proton density fat fraction (MRI-PDFF) as without (< 5%), mild (5–10%), moderate and severe (≥ 10%) steatosis and these cut-off values for discriminating steatosis degree were adopted in the previous clinical trials for estimating effects of different drugs on NAFLD [20–22].

Liver stiffness measurement by real-time shear wave elastography (Super Sonic Imagine, Aix en Provence, France) was performed by two fixed physicians with over 5 years’ experience of ultrasound measurement. A rectangular region of interest (approximately 4 × 3 × 3 cm and set 1–2 cm under the liver surface) was displayed on the best static shear wave elastography image, in which a circular region of interest (the diameter set about 20 mm) without any focal lesion, vessels, biliary tracts, or artifacts from nearby lung gas or cardiac movement) was selected. Then the liver stiffness means, minimum, maximum, and standard deviation (SD) were calculated. The mean value was considered representative of the LSM after five consecutive 2D SWE images were obtained for each patient. The cutoff value of LSM for significant fibrosis was set as over 7.6 kPa that was previously validated in similar subjects [23].

### Statistical analysis

Normally distributed data are presented as the median (standard deviation). The Kruskal–Wallis rank sum test was used for abnormally distributed continuous variables between groups. Pearson’s Chi-squared test was used for comparison of categorical data between groups. Multiple comparisons among groups were performed using ANOVA with Bonferroni post hoc test. Logistic regression models with stepwise selection were used to estimate odds ratios (ORs) for the different stratification of HOMA-IR in relation to metabolic parameters (hypertension, hypertriglyceridemia, high LDL-C level, metabolic syndrome, ALT elevation and AST elevation). We adjusted these models for several potential confounders, including age, sex and BMI. *P* values for trend (two-sided) were calculated. A two-tailed *P*-value less than 0.05 was considered indicative of statistical significance. All data were analyzed using SPSS Statistical software (version 20.0, SPSS Inc., Chicago, IL, USA).

## Results

### Baseline characteristics

Overall, 2768 subjects were enrolled, including 667 in the healthy control (HC) group (24.1%), 970 in the CHB group (35.0%), 878 in the NAFLD group (31.7%), and 253 in the CHB with NAFLD group (9.1%). The baseline characteristics are shown in Table 1. A majority of the study population (approximately 70%) were male, with the median age as 40.1 years. No significant differences in age and sex were found among groups. Compared with the healthy control, all the other three groups presented a somewhat higher waist-to-hip ratio, blood pressure, uric acid, triglyceride and higher total and LDL-cholesterol but not HDL-cholesterol respectively. Metabolic syndrome was most common in the NAFLD group (48.1%), followed by the CHB with NAFLD (31.6%), CHB (7.0%) and HC (1.9%) groups. Furthermore, NAFLD patients with or without CHB tended to have higher BMI, waist-to-hip ratio, blood pressure, uric acid and markedly statistic differences in serum lipid profiles than those healthy control or with CHB alone (Table 1). Notably, higher triglyceride level (2.5 mmol/L vs 1.7 mmol/L, *P *< 0.001) was the only significant differences between the NAFLD group and the CHB with NAFLD group after pairwise comparison. As for HBV viral markers, there was no significant difference regard to HBV DNA level, HBsAg levels and HBeAg positivity rates between CHB patients with or without NAFLD (all *P *< 0.05).

**Table 1:** Baseline characteristics of all patients

	ALL	HC	CHB	NAFLD	CHB with NAFLD
	(N = 2768)	(N = 667)	(N = 970)	(N = 878)	(N = 253)
Gender (male)	1901 (69%)	440 (66%)	651 (67%) ^NS^a^‡^	618 (70%) ^NS^ab	192 (76%) *ab, ^NS^c
Age (year)	40.1 (10.9)	39.3 (9.2)	39.8 (11.7) ^NS^a	41.1 (12.0) ^NS^ab	41.0 (10.2) ^NS^ac, *b
BMI (kg/m^2^)	23.8 (3.9)	21.8 (2.4)	22.2 (3.1) **a	26.5 (3.7) **ab	26.0 (3.7) **ab, *c
Waist–hip ratio	0.85 (0.07)	0.80 (0.06)	0.84 (0.07) **a	0.89 (0.04) **ab	0.89 (0.05) **ab, ^NS^c
SBP (mmHg)	124.2 (13.1)	116.2 (12.1)	124.8 (10.1) **a	127.7 (14.7) **ab	130.0 (10.8) **ab, *c
DBP (mmHg)	79.8 (9.9)	72.1 (9.2)	82.3 (8.1) **a	82.4 (9.9) **a, *b	86.0 (11.1) **ab
CHOL (mmol/L)	5.1 (1.0)	4.7 (0.6)	4.9 (1.1) **a	5.5 (1.1) **ab	5.2 (1.1) **ab, *c
TG (mmol/L)	1.6 (1.7)	1.0 (0.5)	1.3 (0.8) **a	2.5 (2.5) **ab	1.7 (1.3) **ab
HDL-C (mmol/L)	1.3 (0.4)	1.4 (0.2)	1.3 (0.4) ^NS^a	1.1 (0.3) **ab	1.2 (0.4) **ab, ^NS^c
LDL-C (mmol/L)	3.1 (0.9)	2.9 (0.7)	3.1 (0.8) **a	3.3 (0.9) **ab	3.4 (0.9) **ab, ^NS^c
FBG (mmol/L)	5.0 (0.9)	4.8 (0.4)	4.8 (0.7) ^NS^a	5.4 (1.2) **ab	5.3 (1.3) **ab, *c
FINS (μU/mL)	8.7 (6.3)	6.2 (3.4)	7.3 (4.8) *a	11.3 (7.4) **ab	12.0 (7.9) **ab, ^NS^c
HOMA-IR	2.0 (1.8)	1.3 (0.8)	1.6 (1.3) *a	2.7 (2.1) **ab	2.9 (2.6) **ab, ^NS^c
UA (μmol/L)	358.2 (102.4)	309.1 (89.0)	339.0 (92.2) **a	402.8 (102.2) **ab	401.9 (93.2) **ab, ^NS^c
ALT (U/L)	45.9 (63.5)	20.1 (10.9)	55.1 (90.9) **a	51.4 (44.4) **ab	61.5 (64.5) **ab,*c
AST (U/L)	37.1 (49.0)	24.4 (6.0)	44.2 (75.4) **a	37.3 (26.2) **a, *b	44.0 (43.1) **a, *bc
ALB (g/L)	45.6 (3.2)	46.5 (0.2)	44.9 (4.1) **a	45.97 (2.8) **ab	45.2 (3.8) **ac, *b
TB (μmol/L)	14.6 (5.3)	13.6 (0.2)	15.2 (6.8) *a	14.46 (5.1) **a, ^NS^b	14.5 (6.2) *a, ^NS^bc

HC: health control; NAFLD: nonalcoholic fatty liver disease; CHB: chronic hepatitis B; BMI: body mass index; SBP: systolic blood pressure; DBP: diastolic blood pressure; CHOL: total cholesterol; TG: triglycerides; HDL-C: high-density lipoprotein cholesterol; LDL-C: low-density lipoprotein cholesterol; UA: uric acid; ALB: albumin; TB: total bilirubin; ALT: alanine aminotransferase; AST: aspartate aminotransferase; FBG: fasting blood glucose; FINS: serum insulin

Data are n (%) and mean (Standard deviation). *P* values were for the ANOVA analysis across the groups, * *P *< 0.05, ** *P *< 0.001

^‡^a—compared with HC group, b—compared with CHB group, c—compared with NAFLD group, NS— non significant

### Comparison of prevalence and severity of insulin resistance among groups

**Fig. 1:**
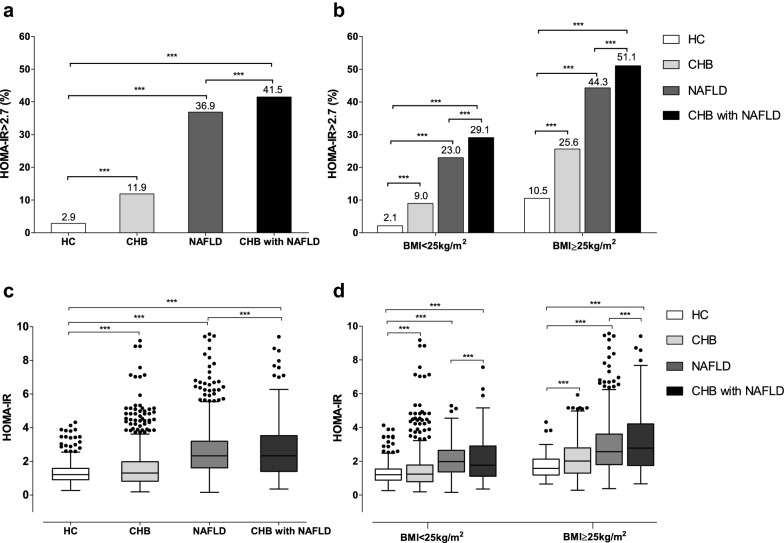
Comparison of HOMA-IR indexes distribution across the body mass index categories in various groups. **a** Proportion of patients whose homeostasis model assessment insulin resistance (HOMA-IR) was higher than 2.7 in health control (HC) group, chronic hepatitis B (CHB) group, nonalcoholic fatty liver disease (NAFLD) group and CHB with NAFLD group, respectively. **b** Proportion of patients whose HOMA-IR > 2.7 with body mass index (BMI) ≥ 25 kg/m^2^ and < 25 kg/m^2^ in HC, CHB, NAFLD and CHB with NAFLD group, respectively. **c** HOMA-IR indexes distribution of patients in HC, CHB, NAFLD and CHB with NAFLD Group, respectively. **d** HOMA-IR indexes distribution of patients with BMI ≥ 25 kg/m^2^ and < 25 kg/m^2^ in HC, CHB, NAFLD and CHB with NAFLD Group, respectively. ***P < 0.001

Compared with the HC group, higher prevalence of IR appeared in all the other groups (*P *< 0.001), with the highest rate in the CHB with NAFLD group (41.5%), followed by NAFLD (36.9%) and CHB (11.9%) (Fig. 1a). It was note that the difference in the proportion of patients with IR was also significant in pairwise comparisons. Likewise, similar trends were observed in the subgroup analysis of obese (51.1% vs 44.3% vs 25.6% vs 10.5%, *P* < 0.05) and nonobese subjects (29.1% vs 23.0% vs 9.0% vs 2.1%, *P *< 0.05) (Fig. 1b).

Additionally, we found that the levels of HOMA-IR in each group was also significantly different, with the highest median HOMA-IR values (75–25% interquartile range) of 2.3 (2.1) seen in the CHB with NAFLD group and followed by 2.3 (1.6) of NAFLD group, 1.3 (1.2) of CHB group and 1.2 (0.7) of HC group, respectively (Fig. 1c). When stratified according to BMI, similar trends existed in obese (1.8 vs 2.0 vs 1.2 vs 1.2, respectively; *P *< 0.05) and nonobese subsets (2.8 vs 2.6 vs 2.0 vs 1.6, respectively; *P *< 0.05).

### Clinical characteristics and differences in contributors to insulin resistance

The clinical characteristics of insulin resistance within each group were listed in Table 2. Across all cohorts (Table 2), subjects with IR presented increased BMI than those without IR. While levels of CHOL, ALT and AST were higher in all groups with IR except for the CHB group, higher SBP was present in each studied group but not in CHB with NAFLD patients, and increased waist-to-hip ratio were observed in all IR population without NAFLD patients. Greater levels of DBP as well as lower HDL-c were found in both patients with IR in healthy controls and CHB group. Within NAFLD groups with or without CHB, elevated LDL-C was shown in those with IR. However, only increased UA concentrations was displayed in healthy control individuals with IR. For all studied groups, no significant differences in ALB and TB were noted.

**Table 2:** Characteristics of all subjects with and without insulin resistance

	HC			NAFLD			CHB			CHB with NAFLD		
	HOMA-IR ≤ 2.7	HOMA-IR > 2.7	*P*	HOMA-IR ≤ 2.7	HOMA-IR > 2.7	*P*	HOMA-IR ≤ 2.7	HOMA-IR > 2.7	*P*	HOMA-IR ≤ 2.7	HOMA-IR > 2.7	*P*
	(N = 648)	(N = 19)		(N = 554)	(N = 324)		(N = 855)	(N = 115)		(N = 148)	(N = 105)	
Male (n %)	430 (66)	10 (53)	0.017	406 (73)	212 (65)	0.318	569 (67)	82 (71)	0.361	111 (75)	81 (77)	0.808
Age (year)	39.4 (9.4)	43.6 (10.3)	0.182	41.4 (11.2)	39.6 (12.2)	0.019	39.9 (11.7)	39.4 (10.3)	0.625	41.2 (10.4)	40.7 (11.1)	0.53
BMI (kg/m^2^)	21.68 (2.39)	23.90 (3.00)	< 0.001	25.67 (3.12)	28.01 (4.22)	< 0.001	21.87 (3.01)	24.21 (3.14)	< 0.001	25.03 (3.03)	27.36 (4.16)	< 0.001
Waist–hip ratio	0.80 (0.06)	0.79 (0.03)	0.001	0.89 (0.04)	0.90 (0.05)	0.557	0.84 (0.07)	0.87 (0.06)	< 0.001	0.88 (0.05)	0.90 (0.05)	0.004
SBP (mmHg)	116.2 (11.8)	124.8 (11.6)	< 0.001	126.8 (13.7)	130.7 (14.7)	0.001	124.2 (10.1)	127.2 (14.0)	0.004	128.7 (10.9)	130.5 (12.0)	0.286
DBP (mmHg)	72.0 (9.4)	74.4 (10.7)	< 0.001	80.9 (10.1)	84.4 (11.0)	0.241	81.1 (7.2)	85.1 (9.7)	< 0.001	85.2 (9.4)	86.7 (12.5)	0.078
CHOL (mmol/L)	4.69 (0.63)	5.18 (0.77)	0.023	5.40 (1.04)	5.57 (1.16)	0.001	4.90 (1.08)	5.03 (1.01)	0.206	5.04 (1.04)	5.53 (1.03)	< 0.001
TG (mmol/L)	0.99 (0.48)	1.33 (0.65)	0.093	2.40 (2.10)	2.69 (2.96)	0.003	1.24 (0.72)	1.56 (1.02)	< 0.001	1.63 (1.04)	1.89 (1.53)	0.114
HDL-C (mmol/L)	1.36 (0.24)	1.31 (0.25)	0.001	1.17 (0.35)	1.09 (0.31)	0.323	1.35 (0.39)	1.24 (0.39)	0.002	1.17 (0.28)	1.17 (0.50)	0.880
LDL-C (mmol/L)	2.87 (0.68)	3.43 (0.78)	0.051	3.28 (0.88)	3.40 (0.93)	< 0.001	3.05 (0.83)	3.16 (0.87)	0.161	3.20 (0.85)	3.64 (0.87)	< 0.001
FBG (mmol/L)	4.77 (0.44)	5.12 (0.51)	< 0.001	5.12 (0.81)	5.79 (1.47)	0.001	4.73 (0.62)	5.56 (0.97)	< 0.001	4.83 (0.65)	5.88 (1.70)	< 0.001
FINS (μU/mL)	5.90 (2.02)	17.86 (11.72)	< 0.001	7.96 (2.58)	16.98 (9.34)	< 0.001	5.96 (2.61)	16.93 (6.00)	< 0.001	7.41 (2.47)	18.37 (8.51)	< 0.001
HOMA-IR	1.26 (0.47)	4.04 (2.71)	< 0.001	1.79 (0.57)	4.31 (2.72)	< 0.001	1.26 (0.59)	4.23 (2.00)	< 0.001	1.59 (0.56)	4.82 (3.04)	< 0.001
UA (μmol/L)	309.4 (88.8)	319.6 (81.2)	< 0.001	394.2 (99.0)	418.9 (106.2)	0.593	337.2 (92.2)	351.9 (89.0)	0.103	393.1 (99.2)	413.0 (83.3)	0.089
ALT (U/L)	20.0 (10.1)	31.0 (17.8)	< 0.001	45.2 (39.2)	61.9 (49.7)	< 0.001	53.5 (91.1)	64.2 (88.3)	0.252	51.9 (55.0)	77.4 (76.2)	0.002
AST (U/L)	23.3 (5.9)	27.4 (6.1)	0.004	35.0 (26.9)	40.2 (24.4)	0.006	44.0 (77.2)	43.9 (48.1)	0.999	40.4 (45.3)	50.9 (40.2)	0.036
ALB (g/L)	46.49 (0.17)	46.54 (0.11)	0.207	46.06 (2.50)	45.81 (3.26)	0.221	44.83 (3.98)	44.96 (4.93)	0.765	45.15 (3.83)	45.21 (3.84)	0.867
TB (μmol/L)	13.59 (0.19)	13.53 (0.14)	0.225	14.62 (4.90)	14.19 (5.31)	0.123	15.28 (6.75)	14.48 (6.83)	0.233	15.44 (7.17)	14.34 (4.46)	0.163

Data are expressed as n (%) and mean (standard deviation)

HC: health control; NAFLD: nonalcoholic fatty liver disease; CHB: chronic hepatitis B; BMI: body mass index; SBP: systolic blood pressure; DBP: diastolic blood pressure; CHOL: total cholesterol; TG: triglycerides; HDL-C: high-density lipoprotein cholesterol; LDL-C: low-density lipoprotein cholesterol; UA: uric acid; ALB: albumin; TB: total bilirubin; ALT: alanine aminotransferase; AST: aspartate aminotransferase; FBG: fasting blood glucose; FINS: serum insulin

Finally, we compared the predictors of insulin resistance between the CHB, NAFLD and CHB with NAFLD groups (Table 3). On multivariate analysis, obesity and hypertension (all *P *< 0.05) were the shared predictors of IR among three groups. Both high LDL-c, low HDL-c and hyperuricemia was independently associated with IR in all the groups except for the CHB with NAFLD group, whereas AST elevation were found to be significantly associated with the presence of insulin resistance in all the groups but not in the NAFLD group. In the CHB group, ALT elevation was the additional factor that was associated with IR prediction (OR: 3.12, 95% CI 1.70–5.76, *P *< 0.001).

**Table 3:** Association of risk factors with insulin resistance in patients with different liver diseases

	CHB				NAFLD				CHB with NAFLD			
	Univariate		Multivariate		Univariate		Multivariate		Univariate		Multivariate	
	*P*	OR	*P*	OR (95% CI)	*P*	OR	*P*	OR (95% CI)	*P*	OR	*P*	OR (95% CI)
Age, years	0.786	1.03	0.83		0.23	0.93	0.38		0.242	0.86	0.76	
Male	0.004	0.45	0.23		0.24	1.22	0.20		1.000	1.01	0.52	
Obesity^a^	< 0.001	4.45	< 0.001	2.97 (1.79–4.94)	< 0.001	3.24	< 0.001	2.80 (1.93–4.05)	< 0.001	2.79	< 0.001	3.23 (1.74–6.00)
Hypertension^a^	< 0.001	3.07	0.001	2.65 (1.48–4.74)	0.001	1.73	0.01	1.56 (1.11–2.20)	0.042	1.84	0.02	2.25 (1.17–4.30)
Hypercholesteremia^a^	0.641	1.148	0.58		0.01	1.50	0.91		0.033	1.81	0.26	
Hypertriglyceridemia^a^	0.001	2.403	0.64		0.08	1.32	0.58		0.828	0.91	0.39	
Low HDL-c^a^	< 0.001	2.56	0.001	2.65 (1.52–4.64)	< 0.001	1.87	< 0.001	2.08 (1.48–2.93)	0.904	1.07	0.33	
High LDL-c^a^	0.015	1.82	0.04	2.14 (1.03–4.45)	< 0.001	1.73	0.01	1.70 (1.14–2.54)	0.022	1.89	0.34	
Hyperuricemia^a^	0.001	2.18	0.03	1.78 (1.07–2.96)	< 0.001	1.75	0.03	1.42 (1.04–1.94)	0.027	1.84	0.28	
ALT elevation^a^	0.002	2.04	< 0.001	3.12 (1.70–5.76)	< 0.001	1.88	0.12		0.003	2.33	0.39	
AST elevation^a^	0.577	1.18	0.02	0.45 (0.24–0.86)	< 0.001	1.98	0.20		< 0.001	3.24	0.004	2.99 (1.41–6.32)
ALB (g/L)	0.495	1.02	0.85		0.24	0.97	0.54		0.931	1.00	0.94	
Total bilirubin (μmol/L)	0.148	0.97	0.12		0.07	0.97	0.22		0.22	0.97	0.15	

Data are expressed as n (%) and mean (standard deviation)

NAFLD: nonalcoholic fatty liver disease; CHB: chronic hepatitis B; ALT: alanine aminotransferase; AST: aspartate aminotransferase; FBG: fasting blood glucose; FINS: serum insulin; ALB: albumin

^a^Obesity was defined as body mass index ≥ 25 kg/m^2^, Hypertension was defined as systolic blood pressure (SBP) ≥ 140 mmHg or diastolic blood pressure (DBP) ≥ 90 mmHg. Hypercholesterolemia was defined as a total CHOL level > 5.2 mmol/L. Hypertriglyceridemia was defined as a TG level > 1.7 mmol/L. A low HDL-C level was defined as an HDL-C level < 1.0 mmol/L. A high LDL-C level was defined as an LDL-C level > 3.4 mmol/L. Hyperuricemia was defined as males and females at > 420 and 360 µmol/L, respectively. The normal upper limit for ALT and AST were set to 40 and 37 U/L, respectively

### Associations of insulin resistance severity and risk of related metabolic abnormalities

**Fig. 2:**
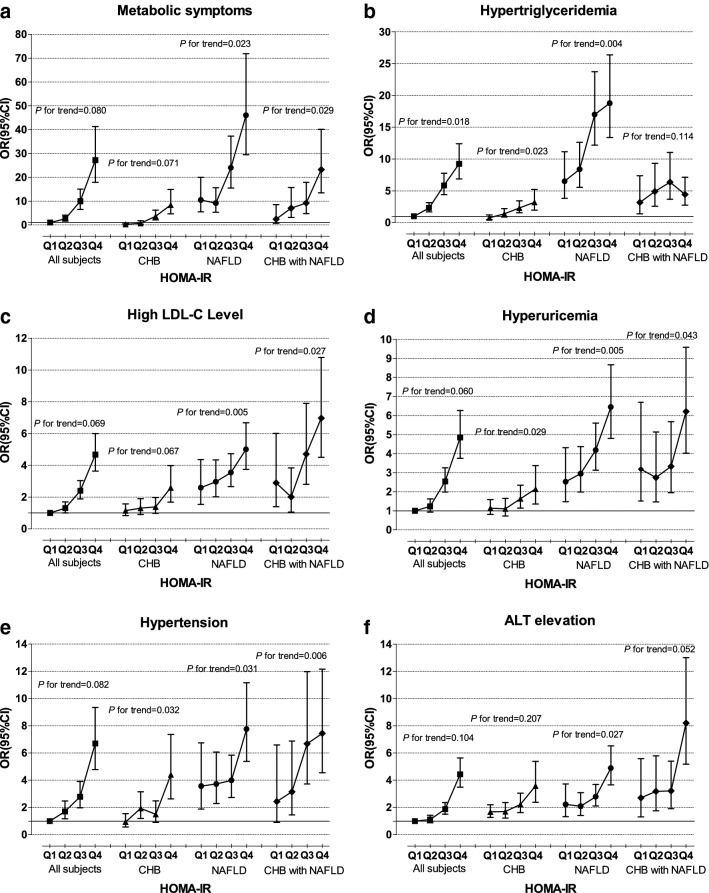
Adjusted odds ratios (ORs) and 95% confidence intervals (CIs), and their trends of varieties of metabolic disorder diseases for quartiles of HOMA-IR. First quartile of HOMA-IR (Q1) in all subjects was set as reference. **a** Hypertension was defined as systolic blood pressure (SBP) ≥ 140 mmHg or diastolic blood pressure (DBP) ≥ 90 mmHg. **b** Hypertriglyceridemia was defined as a TG level > 1.7 mmol/L. **c** High LDL-C level was defined as an LDL-C level > 3.4 mmol/L. **d** Hyperuricemia was defined as males and females at > 420 and 360 µmol/L, respectively. **e** Metabolic symptom was diagnosis as meeting at least three of the following criteria: (1) Waistline > 90 cm (male) and > 80 cm (female) and/or body mass index (BMI) ≥ 25 kg/m^2^; (2) elevated BPs (systolic blood pressure (SBP) > 125 mmHg and/or diastolic blood pressure (DBP) > 70 mmHg); (3) low HDL-C level; (4) fasting serum triglyceride ≥ 1.7 mmol/L; (5) fasting plasma glucose ≥ 5.6 mmol/L. **f** ALT elevation was defined as ALT > 40 U/L

Table 4 presents the risks of related metabolic abnormalities that increased with IR severity (HOMA-IR was categorized by 1.10, 1.60, and 2.70, for the 25th, 50th and 75th percentiles of HOMA-IR, represented by HOMA-IR Q1, Q2, Q3 and Q4). The HOMA-IR Q1 of all subjects was set as a reference. All dose–response relationships were adjusted for age, sex and BMI. Across the quartiles of HOMA-IR levels, there were similar dose–response relationships between IR severity and hypertension (all except for HC, *P* for trend < 0.05, Fig. 2e) and hyperuricemia (all except for HC, *P* for trend < 0.05, Fig. 2d) among all three groups. For metabolic syndrome, no significant influence was presented in CHB patients (*P* for trend = 0.071), but a positive influence was revealed in NAFLD patients (*P* for trend = 0.023, Fig. 2a) and CHB with NAFLD patients (*P* for trend = 0.029, Fig. 2a). A similar dose–response relationship between IR severity and hypertriglyceridemia could be identified in CHB patients (*P* for trend = 0.023, Fig. 2b) and NAFLD patients (*P* for trend = 0.004, Fig. 2b), while such influence disappeared in hyperglyceridemia for CHB patients with NAFLD (*P* for trend = 0.114, Fig. 2b). Regarding LDL-C, HOMA-IR Q4, the highest IR category, presented the highest risk (OR = 6.54, 95% CI 3.65–11.73, *P *< 0.001) in CHB with NAFLD patients, and a clear dose–response relationship was observed between IR and LDL-C in CHB with NAFLD and NAFLD patients; however, no significant positive correlation remained in CHB patients (Fig. 2c). In addition, we observed a significant influence on ALT levels in NAFLD or CHB with NAFLD patients (*P* for trend = 0.207 and 0.042, Fig. 2f) but not in CHB patients (*P* for trend = 0.207, Fig. 2f).

**Table 4:** Association of risk factors for quartile of HOMA-IR in metabolic disorders

	OR (95% CI)^a^			
	All	CHB	NAFLD	CHB with NAFLD
Hypertension				
HOMA-IR Q1	Reference^b^	0.94 (0.57–1.54)	3.57 (1.89–6.75)	2.44 (0.90––6.60)
HOMA-IR Q2	1.72 (1.19–2.50)	1.94 (1.19–3.16)	3.73 (2.29–6.06)	3.15 (1.45–6.87)
HOMA-IR Q3	2.78 (1.98–3.91)	1.50 (0.91–2.48)	3.99 (2.73–5.85)	6.68 (3.73–12.0)
HOMA-IR Q4	6.69 (4.79–9.34)	4.40 (2.64–7.36)	7.75 (5.39–11.2)	7.44 (4.55–12.2)
*P* for trend	0.08	0.03	0.03	0.006
Hypertriglyceridemia				
HOMA-IR Q1	Reference	0.77 (0.49–1.21)	6.52 (3.81–11.1)	3.18 (1.38–7.37)
HOMA-IR Q2	2.31 (1.69–3.16)	1.37 (0.86–2.18)	8.38 (5.56–12.6)	4.90 (2.58–9.34)
HOMA-IR Q3	5.92 (4.45–7.87)	2.29 (1.53–3.42)	17.0 (12.2–23.7)	6.37 (3.68–11.0)
HOMA-IR Q4	9.48 (7.05–12.8)	3.22 (1.98–5.23)	18.8 (13.4–26.4)	4.43 (2.74–7.15)
*P* for trend	0.02	0.02	0.004	0.11
High LDL-c				
HOMA-IR Q1	Reference	1.15 (0.84–1.58)	2.60 (1.54–4.37)	2.90 (1.39–6.01)
HOMA-IR Q2	1.28 (0.98–1.66)	1.31 (0.90–1.90)	2.96 (2.02–4.35)	2.01 (1.06–3.83)
HOMA-IR Q3	2.29 (1.80–2.91)	1.39 (0.98–1.97)	3.55 (2.67–4.73)	4.70 (2.80–7.90)
HOMA-IR Q4	4.64 (3.61–5.97)	2.59 (1.68–3.99)	5.01 (3.75–6.69)	6.97 (4.50–10.8)
*P* for trend	0.07	0.07	0.005	0.03
Hyperuricemia				
HOMA-IR Q1	Reference	1.14 (0.82–1.59)	2.53 (1.48–4.32)	3.19 (1.52–6.70)
HOMA-IR Q2	1.25 (0.95–1.65)	1.10 (0.73–1.66)	2.95 (2.00–4.37)	2.74 (1.47–5.14)
HOMA-IR Q3	2.56 (2.00–3.28)	1.64 (1.14–2.34)	4.19 (3.13–5.61)	3.33 (1.96–5.68)
HOMA-IR Q4	4.96 (3.83–6.42)	2.15 (1.36–3.38)	6.45 (4.80–8.67)	6.21 (4.02–9.58)
*P* for trend	0.06	0.03	0.005	0.04
Metabolic symptoms				
HOMA-IR Q1	Reference	0.27 (0.10–0.80)	10.5 (5.50–19.9)	2.44 (0.70–8.56)
HOMA-IR Q2	2.55 (1.60–4.08)	0.74 (0.30–1.81)	9.15 (5.35–15.6)	7.06 (3.17–15.7)
HOMA-IR Q3	9.62 (6.34–14.6)	3.62 (2.11–6.23)	24.0 (15.5–37.3)	9.17 (4.71–17.9)
HOMA-IR Q4	27.4 (18.0–41.7)	8.34 (4.67–14.9)	46.0 (29.5–71.8)	23.2 (13.4–40.1)
*P* for trend	0.08	0.07	0.02	0.03
High ALT level				
HOMA-IR Q1	Reference	1.68 (1.27–2.21)	2.22 (1.33–3.73)	2.71 (1.32–5.58)
HOMA-IR Q2	1.17 (0.78–1.28)	1.70 (1.22–2.36)	2.10 (1.42–3.09)	3.19 (1.75–5.80)
HOMA-IR Q3	2.01 (1.60–2.53)	2.23 (1.63–3.05)	2.79 (2.11–3.70)	3.22 (1.92–5.40)
HOMA-IR Q4	4.87 (3.82–6.23)	3.59 (2.39–5.39)	4.89 (3.66–6.53)	8.21 (5.18–13.0)
*P* for trend	0.10	0.21	0.03	0.052
Liver fat content (mild vs. moderate and severe)				
HOMA-IR Q1			Reference	Reference
HOMA-IR Q2			1.12 (0.48–3.35)	1.45 (0.12–17.2)
HOMA-IR Q3			2.19 (0.94–5.11)	2.79 (0.33–24.0)
HOMA-IR Q4			10.92 (4.59–25.9)	20.3 (2.49–30.1)
*P* for trend			0.029	0.039

*P* for trend was calculated for the logistic regression analysis tests across the groups

HOMA-IR was categorized by 1.10, 1.60, and 2.70, for the 25th, 50th and 75th percentiles of HOMA-IR, represented by HOMA-IR Q1, Q2, Q3 and Q4

Metabolic symptom was diagnosis as meeting at least three of the following criteria: (1) Waistline > 90 cm (male) and > 80 cm (female) and/or body mass index (BMI) ≥ 25 kg/m^2^; (2) elevated BPs (systolic blood pressure (SBP) > 125 mmHg and/or diastolic blood pressure (DBP) > 70 mmHg); (3) low HDL-C level; (4) fasting serum triglyceride ≥ 1.7 mmol/L; (5) fasting plasma glucose ≥ 5.6 mmol/L. Hypertension was defined as systolic blood pressure (SBP) ≥ 140 mmHg or diastolic blood pressure (DBP) ≥ 90 mmHg. Hypercholesterolemia was defined as a total CHOL level > 5.2 mmol/L. Hypertriglyceridemia was defined as a TG level > 1.7 mmol/L. A low HDL-C level was defined as an HDL-C level < 1.0 mmol/L. A high LDL-C level was defined as an LDL-C level > 3.4 mmol/L. Hyperuricemia was defined as males and females at > 420 and 360 µmol/L, respectively. The normal upper limit for ALT and AST were 40 and 37 U/L, respectively

^a^Adjusted for gender and age

^b^Homeostasis model assessment insulin resistance first quartile (HOMA-IR Q1) of all subjects was set as reference

### Relationship between insulin resistance severity and liver fat content

Among patients with NAFLD and CHB with NAFLD, we further evaluated the relationship between liver fat content and varying degrees of insulin resistance. A total of 644 patients, including 431 NAFLD patients and 213 CHB with NAFLD patients accepted MRI examination. The HOMA-IR Q1 of each group was set as its reference. In NAFLD patients, HOMA-IR Q4 was associated with the highest risk of having moderate and severe steatosis (OR = 10.92, 95% CI 4.59–25.9, *P* for trend = 0.029) after multiple adjustments. Similarly, significant trend was observed as well in CHB with NAFLD patients within HOMA-IR Q4 that a significant influence on having moderate and severe fatty liver through MRI examination (OR = 20.3, 95% CI 2.49–30.1, *P* for trend = 0.039).

## Discussion

In this cross-sectional study conducted in China, we identified different dose–response patterns between insulin resistance and metabolic comorbidities among CHB, NAFLD and CHB combined with NAFLD in 2782 age-matched and gender-matched cases and controls via risk factor analysis. Almost half of the patients with CHB combined with NAFLD had IR, a proportion that was significantly higher than that in other groups with chronic HBV infection or NAFLD alone. We found that increased HOMA-IR was linearly associated with the prevalence of metabolic syndromes in the CHB with NAFLD group. We also found a graded association between the HOMA-IR and uric acid. These associations persisted after multivariable adjustment, including baseline FBG and CHOL concentrations, SBP and BMI. The diverse abovementioned associations remained significant after adjusting for confounders, including family history, age and sex.

Insulin resistance was overrepresented in patients with CHB and NAFLD and to a somewhat greater degree in those who had CHB combined with NAFLD than in the control subjects. Given that insulin resistance contributes to the development of type 2 diabetes, the high prevalence of IR in the CHB group was in line with the previous finding that diabetes is more likely to appear in CHB infection [8–12]. In light of the positive correlation between obesity and insulin resistance, a subgroup analysis stratified by BMI was performed to observe these independent trends. Intriguingly, the proportion of IR and its severity remained elevated in patients with CHB combined with NAFLD compared to those affected by either disease alone. Therefore, a greater risk of IR implies the importance of detecting glucose metabolism at preliminary stages in patients simultaneously affected by CHB and NAFLD.

Insulin resistance has been recognized as a key mechanism of liver steatosis and metabolic morbidity and mortality [7, 24]. When systemic IR exists, the accompanied compensatory hyperinsulinemia over activates insulin receptor-PI3K–Akt signaling in two different organs: liver and adipose tissue [7, 25]. In the former, excess insulin acts as a potent regulator for driving lipogenic genes expression, which leads to over de novo lipogenesis, impaired mitochondrial fatty acid oxidation and subsequent liver inflammation, whereas the latter promotes lipolysis and secret increased levels of adipokines, free fatty lipids and inflammatory cytokines [25]. Therefore, much more toxic lipids were entered, produced, accumulated in liver and released to circulation, causing endothelial dysfunction, hyperlipidemia and related metabolic disorders [7]. The progressively dysregulated metabolism in the liver exacerbate IR in turn, forming positive feedback of worsening metabolism [25]. Although a stepwise increase in IR with the risk of metabolic syndrome was initially reported in NAFLD subjects, the association between IR and CHB with NAFLD was not as well-known as that between IR and NAFLD. Our current results reported that hypertension and increased uric acid show similar trends in CHB with and without NAFLD and first found that IR indicated different clusters of metabolic complications, especially in CHB with NAFLD. No clear trend was found in the association between the fourth quartile of HOMA-IR and hypertriglyceridemia level risk in CHB with NAFLD, while a stepwise increase in IR was associated with an increased risk of hypertriglyceridemia in both CHB and NAFLD. Our findings reinforce those of previous studies demonstrating that HBV infection is negatively associated with hypertriglyceridemia [26] and provide evidence that HBV infection negates the promoting effect of IR on TG levels. One possible mechanism explaining this phenomenon is that HBV products, including HBV protein X, interrupt de-novo lipid synthesis and secretion under insulin-resistant conditions, which are characterized by inhibition of Apo-C3 expression [27] and Apo-B secretion [28]. As the major protein component of triglyceride (TG)-rich lipoproteins, decreased Apo-B and Apo-C3 concentrations therefore lower serum very-low-density lipoprotein (VLDL) and TG levels.

Several studies reported the inverse association of HBsAg positivity with total cholesterol and LDL-C. In a large cross-sectional study conducted in a Taiwanese cohort with 56,336 participants [29], patients with positive HBsAg had a significantly lower frequency of hypercholesterolemia. Additionally, a longitudinal study further demonstrated an inverse association between HBV infection and dyslipidemia occurrence over time [30]. Notably, IR exhibited a positive association with higher LDL-C levels in CHB with NAFLD than in CHB or NAFLD alone in our study. Our findings suggested that concomitant steatosis during HBV infection modifies the association between IR and LDL-C. A higher HOMA-IR index might serve as a predictive factor for the prevalence of hypercholesterolemia. Although the underlying mechanism needs further exploration, the combination of HBV infection and NAFLD appears to have an opposite effect on liver cholesterol metabolism from that on TG metabolism.

Insulin resistance was identified as a driver of hepatic steatosis progression [31]. IR severity has been proven to be significantly associated with the degree of steatosis in NAFLD. The present study observed similar associations with increased HOMA-IR and intrahepatic triglyceride (IHTG) content in both NAFLD and CHB with NAFLD. Moreover, we also observed a greatly increased prevalence of elevated ALT levels in CHB with NAFLD compared with those in NAFLD after adjusting for compounding factors. This finding suggests that HBV infection may have a synergic effect with IR on liver damage. In addition to chronic HBV-infection-induced inflammatory response and lipotoxicity caused by steatosis in hepatocytes, IR predisposes cells to reactive oxygen species generation and lipid peroxidation [7], and thus, these factors contribute to liver injury aggravation. Liver damage is accompanied by increased production of proinflammatory cytokines, which are, in turn, involved in IR.

Our findings strongly highlight the high risk of IR occur in both NAFLD, CHB and their combination, and the necessity for initiating screening and intervention. Without cure for IR by drugs, combination of dietary modification and intensified exercise training remained the cornerstone in the management of IR and its comorbidities [32]. Adjusting the calories, ratio of carbohydrate, fatty acid and proteins in the diet have both been advocated as dietary strategies to improve insulin sensitivity. There is emerging evidence that Mediterranean-style diet, hypocaloric low-carbohydrate or low-fat diet (by energy deficit of 500–750 kcal/day), and low-glycaemic index (GI) diet confer improved weight control and metabolic profiles among insulin resistance, metabolic syndrome and NAFLD patients [32, 33]. An individualized exercise intervention, such as high-intensity interval training, moderate-intensity for 150 min/weeks or 75 min/weeks of vigorous-intensity exercise, may also provide an additional therapeutic effect on insulin resistance [34]. Therefore, our results evaluating the effect of IR on varied metabolic disorders provided the evidence base for individualized dietary and exercise recommendations on modifying insulin sensitivity to reduce progression to related metabolic diseases.

Our study had some limitations. First, we did not apply the euglycemic clamp technique, the gold standard of IR measurement, for analysis. The euglycemic clamp technique is limited in our large population study because of its cost and complexity. Thus, the conclusion that the relationships among insulin resistance and metabolic alteration across different liver diseases needs further study. Second, we conducted a cross-sectional study based on the Chinese population, and we need a larger sample of long-term follow-up data in the future to support our conclusions. Furthermore, assessing the impact of viral factors on IR severity and their interactions with metabolic profiles would be interesting. Third, other confounders related to metabolic parameters, such as dietary habits, were not available, and we could not adjust for such confounders and further explored the potential benefit of modulating insulin resistance through individualized nutritional intervention.

## Conclusion

Chronic hepatitis B with NAFLD patients have a higher prevalence of insulin resistance, and a high burden of insulin resistance is associated with an increased risk of related metabolic disorders (total cholesterol, LDL-cholesterol, hypertension and high uric acid but not triglycerides) and liver fat content. Although our study was a cross-sectional analysis, this is the first time we used a dose–response trend analysis method to estimate the relationships between IR severity at diagnosis and related metabolic disorders among subjects with CHB, NAFLD and CHB with NAFLD. These findings in our study add to existing evidence that IR meditates different extents of metabolic abnormalities in different liver diseases, suggesting that in patients with CHB combined with NAFLD, insulin resistance and related metabolic disorders need intensive management.

## Data Availability

The datasets used and/or analysed during the current study are available from the corresponding author on reasonable request. All data generated or analysed during this study are included in this published article.

## Abbreviations

NAFLD: nonalcoholic fatty liver disease
CHB: chronic hepatitis B
HC: health control
HBV: hepatitis B virus
IR: insulin resistance
HOMA: homeostasis model assessment
HBsAg: hepatitis B surface antigen
CT: computed tomography
MRI: magnetic resonance imaging
MRI-PDFF: MRI proton density fat fraction
LSM: liver stiffness measurement
BMI: body mass index
WHR: waist-to-hip ratio
SBP: systolic blood pressure
DBP: diastolic blood pressure
FBG: fasting blood glucose
FINS: serum insulin
CHOL: total cholesterol
TG: triglycerides
HDL-C: high-density lipoprotein cholesterol
LDL-C: low-density lipoprotein cholesterol
UA: uric acid
ALB: albumin
TB: total bilirubin
ALT: alanine aminotransferase
AST: aspartate aminotransferase
SD: standard deviation
